# Development and Preliminary Validation of the Turkish Prosodic Comprehension Test (PCT)

**DOI:** 10.3390/audiolres16040099

**Published:** 2026-06-30

**Authors:** Merve Savaş, Göknur Miray Ceyhan Tasin, Senanur Kahraman Beğen, Melis Buse Arslan, Ayşe Nur Koçak, Tutku Altıntaş

**Affiliations:** Department of Speech and Language Therapy, Faculty of Health Sciences, Istanbul Atlas University, Istanbul 34408, Turkey; g.m.ceyhann@gmail.com (G.M.C.T.); senanur.kahraman@atlas.edu.tr (S.K.B.); melis.altun@atlas.edu.tr (M.B.A.); aysenur.kocak@atlas.edu.tr (A.N.K.); tutku.altintas@atlas.edu.tr (T.A.)

**Keywords:** prosodic comprehension, prosody, test development, Turkish language, reliability, validity

## Abstract

**Background:** Prosodic cues play a critical role in marking syntactic boundaries and guiding sentence interpretation. However, Turkish clinical language batteries lack dedicated measures targeting linguistic prosody and the syntax–prosody interface. Consequently, subtle auditory–prosodic comprehension difficulties may go undetected in stroke populations who perform within normal limits on standard aphasia assessments. This study presents the development and initial psychometric evaluation of the Prosodic Comprehension Test (PCT), a Turkish sentence–picture matching tool designed to isolate prosodic contributions to meaning under controlled syntactic conditions. **Methods:** A total of 440 neurologically healthy native Turkish-speaking adults participated. An initial pool of 80 sentences (40 minimal pairs), identical in segmental and syntactic structure but differing in interpretation through prosodic boundary placement, was created. Audio stimuli were recorded by a professional actor, and corresponding visual stimuli represented alternative interpretations. Following expert review, 32 sentences (16 pairs) were retained and organized into three subcomponents: In situ Prosody, Focus–Topic Marking, and Pragmatic Disambiguation. Administration included fixed-intensity auditory presentation and a structured learning phase. **Results**: Internal consistency was acceptable (Cronbach’s α = 0.73). Principal component analysis was consistent with the theoretically proposed three-component structure (KMO = 0.74; Bartlett’s test significant, *p* < 0.001), with the three components collectively accounting for 28.2% of the total variance. Convergent validity was supported by a significant positive correlation with MoCA-TR (r = 0.23, *p* < 0.001, 95% CI [0.14, 0.32]). **Conclusions:** The PCT appears to be a linguistically grounded and psychometrically promising tool for assessing prosodic comprehension in Turkish. The present findings are based on a healthy adult sample and should be interpreted as preliminary normative evidence. Further research should address test–retest reliability, confirmatory factor analyses, and validation in clinical populations.

## 1. Introduction

### 1.1. Clinical Gap in Auditory–Prosodic Assessment

Prosodic cues play a central role in signaling syntactic boundaries and shaping sentence interpretation in spoken language [1]. For the purposes of this study, prosodic comprehension is defined as the ability to use prosodic boundary placement and prominence cues—including pauses, prolongations, and pitch changes—to derive sentence meaning at the levels of syntactic structure, information structure (focus–topic relations), and pragmatic disambiguation. This definition encompasses three partially dissociable mechanisms that informed both the conceptual design and the factor structure of the PCT: (a) sensitivity to prosodic boundary placement in in situ structures; (b) the use of prosody to signal focus–topic relations within a fixed syntactic frame; and (c) prosody-driven disambiguation in contexts involving morphological ambiguity, homophony, or underspecified referential relations.

Existing Turkish-language clinical assessment tools do not include dedicated measures targeting linguistic prosody or the syntax–prosody interface. A growing body of evidence shows that multiple components of language may become impaired in stroke patients even in the absence of aphasia, including those with cerebellar or right-hemisphere lesions [2]. Consequently, subtle auditory–prosodic comprehension impairments may remain undetected in stroke populations who otherwise perform within normal limits on standard aphasia assessments [3]. This persistent gap underscores the necessity of developing a language-specific assessment instrument capable of isolating prosodic contributions to sentence interpretation with methodological precision. Accordingly, the primary motivation of the present study was to develop an auditory comprehension instrument that explicitly incorporates prosodic processing demands, enabling the detection of subtle comprehension impairments that remain invisible to existing clinical tools. To ensure its applicability in clinical settings, the Prosodic Comprehension Test (PCT) was first evaluated for reliability and construct validity in healthy adults.

### 1.2. Neurocognitive Basis of Prosodic Processing

According to the Dual Stream Model, although the left hemisphere remains central to language processing, the right hemisphere, together with subcortical structures and widespread axonal pathways, is also critically involved in auditory comprehension and speech recognition. This model highlights the existence of bihemispheric functioning from the early phases of auditory perception. The auditory comprehension process, which begins with spectrotemporal analysis in the primary auditory cortices of both the left and right hemispheres, results in the production of a holistic speech-recognition output through white matter pathways leading to the more advanced auditory processing cortices in both hemispheres [4]. In this process, the right and left hemispheres integrate different physical elements of auditory stimuli: the right hemisphere processes pitch information (spectral analysis), while the left hemisphere processes short-term auditory stimuli with narrower temporal ranges (rapid temporal processing) [5].

In auditory comprehension, the boundaries of syntactic units such as words, phrases, and clauses are identified by perceiving multiacoustic cues in an ongoing speech signal. These syntactic units, parsed through the processing of prosodic elements such as pauses, prolongations, and changes in pitch, enable the comprehension of alternative meanings and language-specific metaphorical or abstract meanings that the utterance may have. The semantic possibilities created by the syntax–prosody interaction are interpreted in accordance with the characteristics of the context in which the language is spoken (e.g., **Uyuz**, Ahmet’e geldi [Ahmet is infected with scabies] vs. **Uyuz Ahmet’e**, geldi [He came to the idiot Ahmet]) [1,6].

Prosody refers to changes in the physical properties of the speech signal, such as pitch, rhythm, and intonation, which convey communicative goals, emotions, and attitudes [7]. Prosody, as one of the suprasegmental features of oral communication, is critically important for interpersonal interaction [8]. Emotional and linguistic prosody is a function of a large-scale neurocognitive network, mostly lateralized to the right hemisphere, but also includes analogous areas in the left hemisphere [9,10]. In the perception of linguistic prosody, activity is observed in anterior superior temporal areas in both hemispheres, the inferior frontal gyrus and intraparietal sulcus in the right hemisphere, and the frontal operculum in the left hemisphere [11]. Tone changes emphasizing pauses or sentence-final intonation are predominantly processed in the right hemisphere [12], while semantic-lexical syllable stress is processed in the left hemisphere [13].

The distinction between declarative and interrogative sentences, whose prosodic elements are typically processed by the right hemisphere, may not apply to Turkish. In Turkish, interrogative sentences are formed by using question suffixes and/or question words. While interrogative sentences without question words and affixes are rare, they do occur in certain Anatolian dialects [14].

The widely accepted view in language research is that the left hemisphere is dominant in morphosyntactic processing [15], semantics [16], and linguistic prosody, while the right hemisphere is involved in processing the pragmatic dimension of language and emotional prosody [17]. Therefore, affective prosody is impaired in right hemisphere damage, whereas linguistic prosody is more affected by left hemisphere damage [18]. However, recent research shows that the prosodic function of the right hemisphere is not limited to emotion, as right-lateralized activity in the dorsolateral prefrontal cortex occurs during the production of linguistic prosody [19]. The basal nuclei and cerebellum are also involved in the perception and production stages of linguistic prosody [20]. Similarly, emotional prosodic perception and production may be impaired with left hemisphere damage [21], and prosodic discrimination skills for perceiving the boundaries of clusters may be impaired in individuals with both left and right hemisphere lesions [22].

### 1.3. Linguistic Prosody in Turkish

Turkish, an agglutinative language characterized by hierarchically arranged suffixes and obligatory phonological harmony, relies heavily on dependent morphemes, which are critical both semantically and morphosyntactically. In addition, a lexical root that has not received any inflectional or derivational suffix can function as both a syllable and a word. Due to the prosody of Turkish, stress is typically placed on the last syllable of words. As inflectional or derivational suffixes are added to the root, the stress shifts toward the last suffix added (e.g., Araba [car], arabada [in the car], arabadaki [the one in the car], arabadakiler [the ones in the car]).

An example of linguistic prosodic production can be observed in the way speakers shift syllable stress to distinguish between homophones, that is, words that share the same orthographic form but differ in meaning. For instance, the word Ordu may refer either to a province in Turkey or to the concept of an army, and sentences such as Ahmet kapıyı açtı (“Ahmet opened the door”) and Ahmet’in karnı açtı (“Ahmet was hungry”) illustrate how prosodic stress placement differentiates otherwise identical surface forms. Furthermore, the syllables of a homophonic word are stressed differently depending on whether it serves a different grammatical role in the sentence, such as an adjective or adverb (e.g., Artık [adjective] vs. Artık [adverb]) [23]. Turkish has a flexible and rich structure regarding linguistic prosodic elements; emphasis can be placed on syllables, words, or word groups depending on contextual conditions [24]. Turkish is a permutable, transpositive language, meaning that word order in a sentence structure can be changed based on how the flow of the idea is expressed. In Turkish, the element to be emphasized is usually placed immediately before the predicate or brought closer to it (e.g., Ayşe Boston’a uçakla gitti [Ayşe went to Boston by plane, not by car], Ayşe uçakla Boston’a gitti [Ayşe took a plane to Boston, not to New York]).

Even when the syntax is fixed, syllable stress can shift to reflect the semantic context. The study of linguistic prosodic elements in Turkish requires a comprehensive design that cannot be contained within a single study, due to factors such as the flexibility of syntax based on the communication context and the language’s richness in homophones [25]. Studies examining linguistic prosody deficits following acquired brain damage have frequently focused on the contribution of prosodic cues to the comprehension of syntactic phrasing or information structure, commonly referred to as the syntax–prosody interface [26].

### 1.4. Information Structure, Syntax–Prosody Interface, and Contextual Effects

Many studies on the expression of information structure through prosody and syntax in Turkish emphasize the central role of communicative context. There is broad agreement that focus, which conveys new information, is typically positioned immediately before the verb. Topic, which represents information shared between the speaker and the listener, is generally placed at the beginning of the sentence. However, analyses of Turkish sentence datasets demonstrate that discourse context can itself be shaped prosodically, and that the syntactic position of the focused element may show considerable flexibility [27,28,29]. In addition, the element intended to be highlighted in Turkish sentences can be pragmatically marked in situ through prosodic boundary placement, even when it is not moved to sentence-initial position or placed immediately before the verb [30].

Evidence from a discourse corpus of 1152 Turkish sentences drawn from 1144 spoken dialogues shows that the formation of intonational phrases is influenced by contextual factors such as the speaker’s communicative goals and intentions [31]. Furthermore, the high degree of morphological productivity in Turkish affects both morphosyntactic and prosodic word structure. A written corpus study of 200 million Turkish words revealed that nearly 34 million tokens exhibit morphological ambiguity [32]. In natural discourse, pragmatic interpretation frequently depends on prosodic boundary placement, particularly in contexts involving morphological ambiguity, homophony, or underspecified referential relations. Prosody therefore functions as a primary cue for pragmatic disambiguation.

In line with these linguistic properties, the pragmatic disambiguation subscale of the PCT was constructed using a larger and more diverse pool of items. This structure reflects the naturally occurring ambiguities in Turkish that arise from the interaction between context and prosodic boundary placement. The expanded item pool enables the instrument to capture not only prosodic contributions to syntax–prosody mapping but also the pragmatic inferencing processes that are fundamental to everyday Turkish comprehension.

By incorporating sentences that involve homophony, morphological underspecification, and referential ambiguity, all of which are widely attested in Turkish discourse, the PCT provides an assessment framework that reflects the types of interpretive challenges commonly encountered in natural language use.

Taken together, these linguistic and pragmatic characteristics of Turkish indicate that prosodic comprehension relies on several partially dissociable mechanisms. These include the ability to parse syntactic structure through prosodic boundary placement, the use of prosody to signal relations between focused and topical elements, and the interpretation of prosodic cues to resolve pragmatic ambiguity. Each of these mechanisms informed both the conceptual design and the factor structure of the PCT.

### 1.5. Stroke, MCA Lesions, and Subthreshold Language Impairment

The middle cerebral arteries supply core anatomical structures associated with language, including the lateral surfaces of the cerebral hemispheres, the internal capsule, various thalamic nuclei, the basal ganglia, and associated white matter pathways; accordingly, lesions in the middle cerebral artery (MCA) territory are known to cause language disorders [33]. The incidence of stroke in Türkiye is steadily increasing, affecting approximately 0.14% to 0.16% of the population [34]. Ischemic stroke most frequently occurs in MCA territory, accounting for approximately 45.7% of cases [35].

The literature indicates that linguistic prosody can be impaired at both comprehension and production levels following brain damage in either hemisphere, although there is no definitive consensus regarding lateralization [26]. Standard assessment tools used for evaluating acquired language impairments in Turkish do not take into account prosodic elements in auditory sentences [36,37,38,39]. Communication difficulties significantly impair the quality of life in individuals with aphasia in Turkey [40]. Similarly, even non-aphasic stroke patients scoring within the normal range on language subtests may experience difficulties in social and familial relationships [41], suggesting that standard tools may fail to detect subthreshold language impairments. These observations motivate the development of a dedicated prosodic comprehension measure, though the present study is limited to healthy adults and does not yet establish clinical diagnostic validity.

### 1.6. Rationale for the Prosodic Comprehension Test (PCT)

Prosody functions as a fundamental component of verbal communication, mediating structural, pragmatic, and cognitive–affective information [42]. In Turkish, prosodic boundary placement plays a particularly important role in sentence interpretation due to the language’s flexible word order, rich morphological structure, and frequent use of context-dependent ambiguity. Despite these linguistic characteristics, existing Turkish-language assessment tools do not include dedicated measures targeting linguistic prosody or the syntax–prosody interface. Accordingly, the primary aim of the present study was to develop a linguistically grounded instrument capable of assessing prosodic comprehension in Turkish under controlled syntactic conditions. The PCT was designed to evaluate how prosodic cues contribute to sentence interpretation across syntactic structure, information structure, and pragmatic disambiguation. The development process included the generation of prosodically manipulated sentence pairs, expert review procedures, and preliminary psychometric evaluation in a neurologically healthy adult sample. Given the increasing interest in subtle language disturbances that may not be captured by conventional language assessments, the development of dedicated prosodic comprehension measures may also contribute to future clinical research investigating auditory–prosodic processing in neurological populations. However, the present study is limited to test development and preliminary normative evaluation in healthy adults.

## 2. Materials and Methods

### 2.1. Participants

Sample size (*N* = 440) was determined based on anticipated reliability requirements and previous large-scale normative studies in the literature, which recommend large samples to ensure representativeness and stability of estimates [43]. All individuals scored 26 or above on the Turkish version of the Montreal Cognitive Assessment (MoCA-TR) and had no history of primary neurological or psychiatric disorders. Participants were native Turkish speakers, recruited from the general community. Participants provided written informed consent prior to participation. Ethical approval was obtained from the Istanbul Atlas University Non-Interventional Clinical Research Ethics Committee (Date: 23 March 2026; Meeting No: E-22686390-050.99-96206).

Table 1 describes the demographic characteristics of the participants, including educational level, language background, and gender distribution.

### 2.2. Instruments

#### 2.2.1. Montreal Cognitive Assessment Turkish Version (MoCA-TR)

MoCA-TR is a brief cognitive screening tool developed to detect mild cognitive impairment and early dementia. It evaluates multiple cognitive domains, including attention, executive functions, memory, language, visuoconstructional skills, conceptual thinking, calculation, and orientation. The total score ranges from 0 to 30, with higher scores indicating better cognitive performance. A score of 26 or above is typically considered within the normal range [44]. In the present study, MoCA-TR was administered as a standardized measure of global cognitive functioning to support convergent validity evidence for the PCT, that is, to examine whether prosodic comprehension performance is associated with general cognitive status in a healthy sample. MoCA-TR is not used here as a criterion reference standard for prosodic processing.

#### 2.2.2. Development of the PCT

It is known that standardized tools currently used to assess language impairment in Turkish-speaking individuals following cerebrovascular injury do not include specific subtests targeting prosodic comprehension [36,37,38,39]. To address this gap, an initial item pool was created consisting of 80 sentences (40 pairs) with identical syntactic structures, where the semantic interpretation varied primarily based on prosodic changes. These items were specifically designed to reflect Turkish prosodic patterns that alter grammatical class or pragmatic meaning while preserving syntactic surface form.

Each sentence was audio-recorded by a professional male actor using natural prosodic variations corresponding to the intended meaning. Accompanying visual stimuli (illustrative images) were created by an artist and selected by the researchers to represent the two different interpretations of each sentence, thereby supporting comprehension during task administration. The images were chosen to be culturally and contextually appropriate, with clarity and simplicity prioritized to avoid bias.

To ensure content validity, the 80 sentences—along with their corresponding audio and visual materials—were submitted to expert review by a panel consisting of one neuropsychologist, two neurologists, and two speech and language therapists. Based on expert feedback regarding prosodic clarity, linguistic naturalness, and semantic distinctiveness, 32 sentences (16 pairs) were selected for inclusion in the final version of the PCT. Each expert independently rated every candidate item on prosodic clarity, linguistic naturalness, and semantic distinctiveness using a 4-point scale. An item was retained when at least four of the five experts rated it as acceptable. Disagreements were resolved through panel discussion until consensus was reached, and items flagged by the reviewers were revised or removed accordingly before the final 32-item set was established. Sentences excluded from the final set were eliminated for reasons such as the impossibility of concrete visualization, semantic implausibility or awkwardness, and the presence of orthographic elements. It should be noted that while the expert review process addressed prosodic clarity and content validity, a fully controlled acoustic analysis confirming the absence of non-prosodic cues across all stimuli was not conducted in the present study and constitutes an area for future work. The selected items and their corresponding stimulus materials are presented in Table 2.

As an additional transparency check, an illustrative acoustic inspection was conducted for one representative item pair, *Balkona çıktı* (“He went out to the balcony”) and *Balkon*, *açıktı* (“The balcony was open”). The results are provided in Supplementary Table S1. This analysis was included only as a descriptive example of how prosodic boundary placement may be acoustically reflected in one item pair and was not intended to replace systematic acoustic validation of the full stimulus set.

The PCT was designed to isolate prosodic contributions to meaning through a sentence–picture matching paradigm. Listeners are presented with visual stimuli to engage the strong influence of pragmatic factors on prosodic prominence and intonation in Turkish and to constrain the context in a way that facilitates accurate responses. To neutralize the effects of Turkish’s flexible word order, the PCT employs syntactically identical sentences in Subject-Object-Verb (SOV) order, in which meaning distinctions arise primarily through prosodic boundary placement.

The PCT includes items that target three complementary components of prosodic comprehension in Turkish: (a) sensitivity to prosodic boundary placement in in situ structures; (b) the use of prosody to mark focus–topic relations within a fixed SOV frame; and (c) pragmatic disambiguation in contexts involving morphological or referential ambiguity. In the PCT, listeners are always presented with two images corresponding to two plausible interpretations of the same sentence. Each item was designed to emphasize a primary prosodic contrast and its interpretive consequence.

Items were categorized into three theoretically motivated subcomponents. The In Situ Prosody subscale (Items 1, 3, 9, 11, 13, 15, 19, and 21) comprised items in which prosodic boundary placement does not alter the semantic interpretation and the target response corresponds to the neutral, structurally default parse. The Focus–Topic Marking subscale (Items 2, 4, 10, 12, 14, 16, 20, and 22) included items in which prosodic prominence modulates information structure by assigning contrastive focus or topicalization, shifting the intended meaning. The Pragmatic Disambiguation subscale (Items 5–8, 17–18, 23–32) consisted of items in which interpretation relies on prosody-driven pragmatic inference, typically involving morphological ambiguity, homophony, or context-sensitive referential contrast.

Sentence pairs have the same syntactic structure, but depending on prosodic change, the grammatical class of the word or phrase changes and the subject of the surface structure may become latent. Since Turkish obligatory subject-predicate agreement allows the subject to be inferred from the predicate, null subjects are possible [45].

(1) *Yaşlı, ağacın altında uyuyordu. ‘The old man was sleeping under the tree.’ The old man tree-GENITIVE under-3.SINGULAR POSSESSIVE-LOCATIVE sleep-IMPERFECTIVE-3.SINGULAR.*
(2) *Yaşlı ağacın, altında uyuyordu. ‘He was sleeping under the old tree.’ He (null subject) the old tree-GENITIVE under-3. SINGULAR.POSSESSIVE-LOCATIVE sleep-IMPERFECTIVE- 3.SINGULAR.*

**Figure 1:**
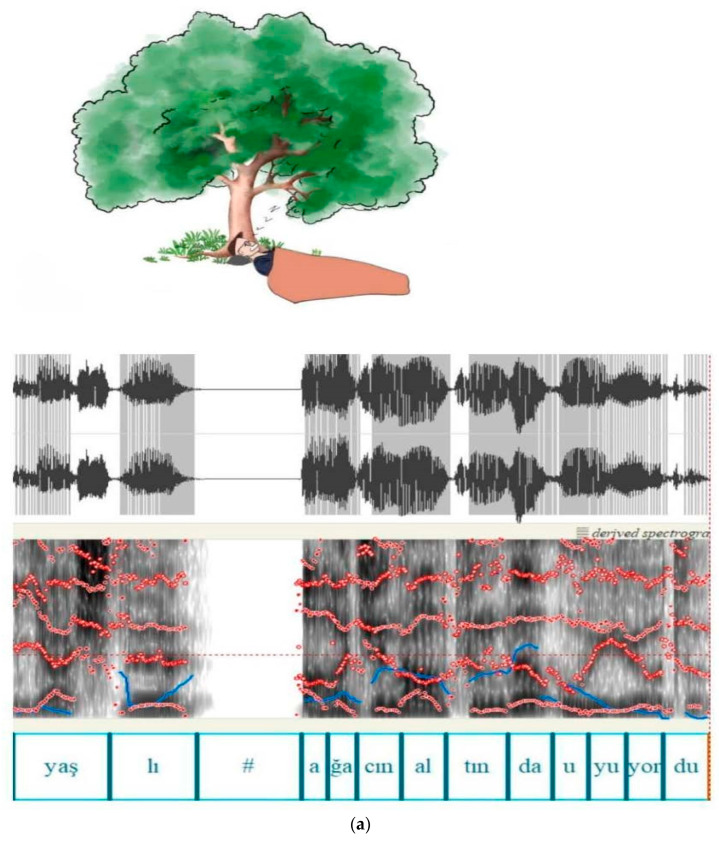

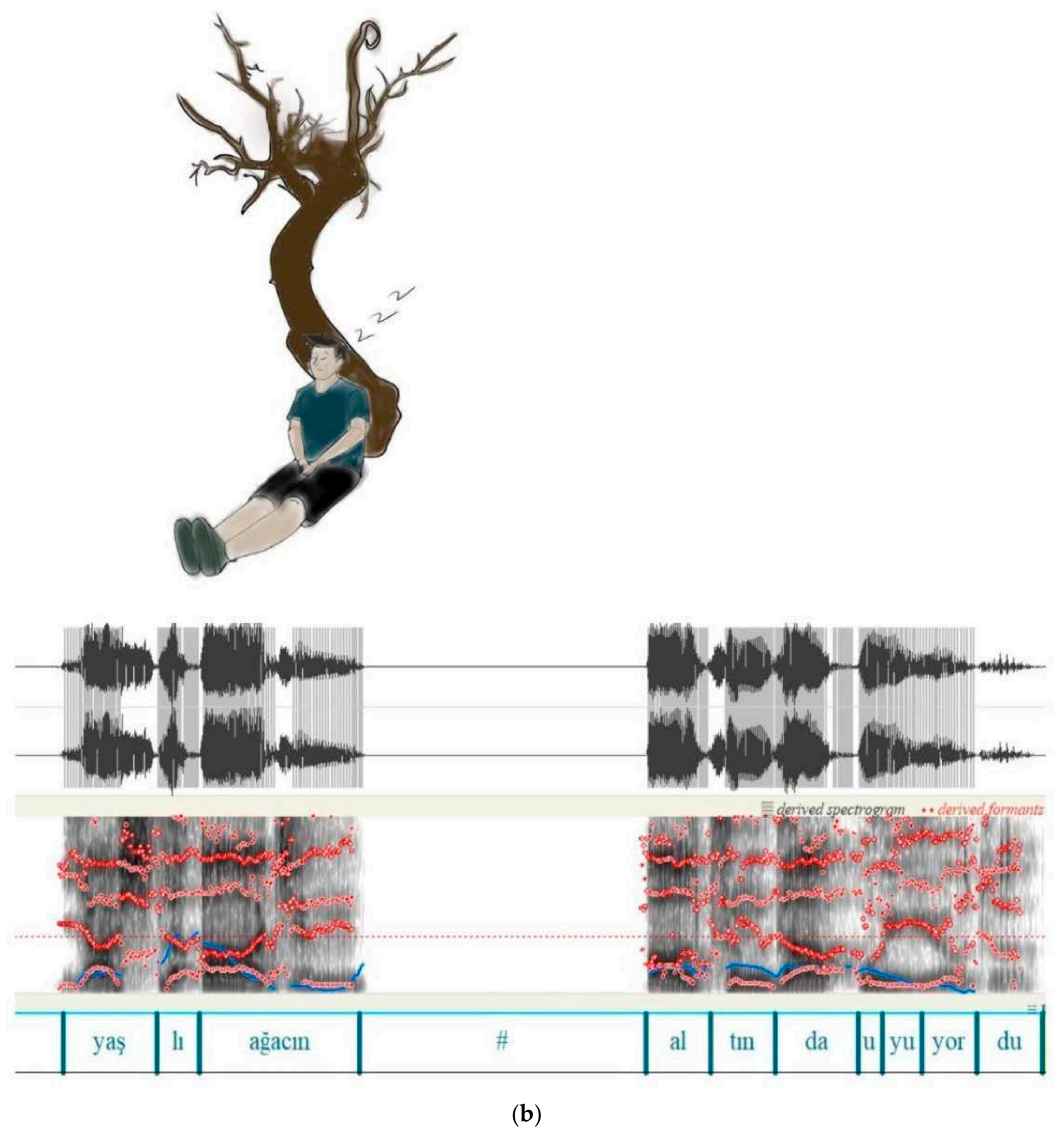
Examples of PCT items. (**a**) Yaşlı, ağacın altında oturuyordu. (**b**) Yaşlı ağacın, altında oturuyordu.

In Sentence (1) (in situ condition) (Figure 1a), the prosodic boundary placed immediately after *yaşlı* signals that *yaşlı* functions as a nominal head referring to an elderly human referent. The syntactic subject (“the old man”) is overtly present in the surface structure, and the predicate phrase (“was sleeping under the tree”) is interpreted accordingly. Structurally, *yaşlı* projects as the head of a Determiner Phrase (DP) referring to an agentive individual. This DP can be represented as [DP [D° Ø] [NP [AdjP yaşlı] [N° (adam)]]].

The phrase *ağacın altında* functions as a locative Prepositional Phrase (PP) specifying the spatial setting of the event. Thus, the prosodic boundary in (1) supports an in situ, agentive interpretation by maintaining the default DP structure. In Sentence (2) (focus–topic condition) (Figure 1b), the prosodic boundary follows the string *yaşlı ağacın*, forcing a head–modifier reanalysis. Here, *yaşlı* is reinterpreted as an attributive adjective modifying *ağaç*. The nominal phrase becomes: [DP [D° Ø] [NP [AdjP yaşlı] [N° ağaç]]].

This DP is genitive-marked (yaşlı ağacın) and forms part of a locative adjunct. The agent of the verb is therefore reanalyzed as a null subject, whereas *yaşlı ağaç* becomes the possessor within the PP. As a result, the sentence describes sleeping under the old tree, not sleeping by an old man. These two prosodic contours thus correspond to distinct syntactic structures and yield sharply divergent interpretations despite identical segmental content.

(3) *Balkona çıktı. ‘He went out to the balcony.’ He (null subject) balcony-DATIVE went.out-PAST-3.SINGULAR.*
(4) *Balkon, açıktı. ‘The balcony was open.’ Balcony open-PAST COPULA-3.SINGULAR.*

**Figure 2:**
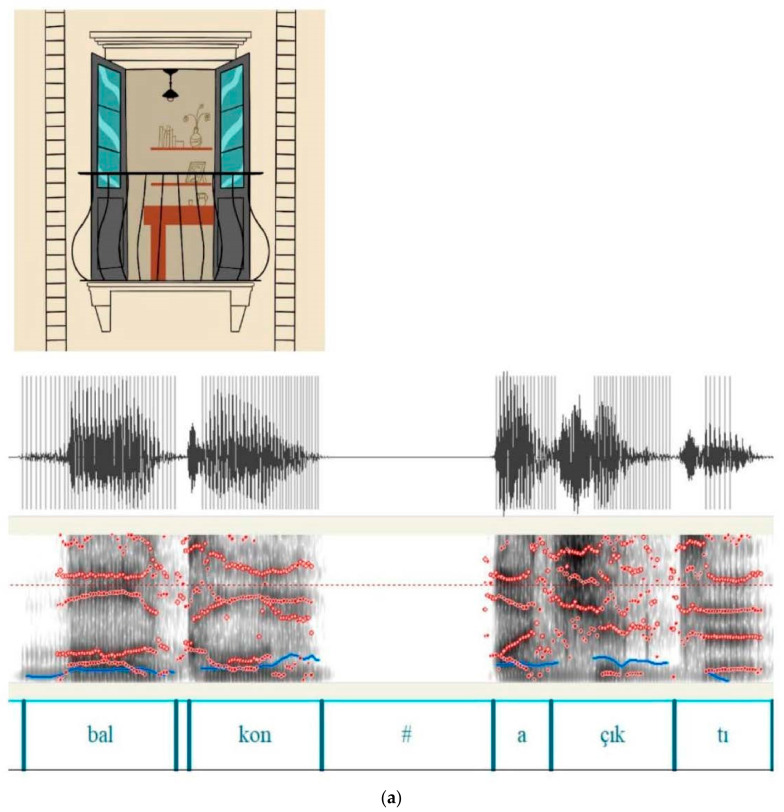

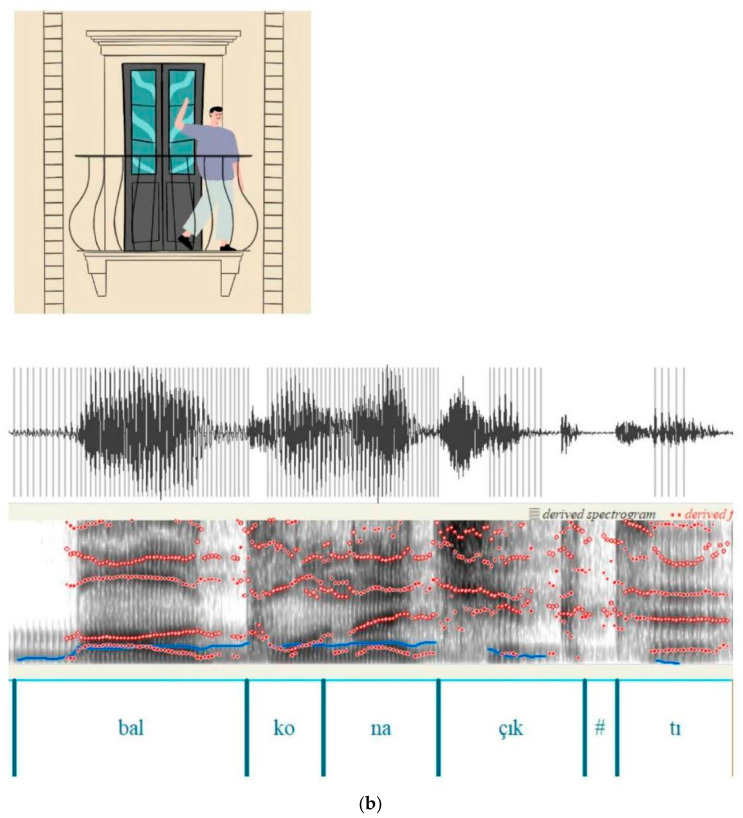
Example of PCT item. (**a**) Balkon açıktı. (**b**) Balkona çıktı.

In Sentence (3) (Figure 2a), the subject is null and interpreted as the agent of the motion event. The dative-marked form *balkona* functions as the goal argument of the verb *çıkmak*. The entire DP *balkon-a* is parsed as part of a PP complement to the verb, represented as [vP [DP pro] [v’ [VP [PP [DP balkon-a]] çık-tı]]]. Crucially, the absence of a prosodic break ensures that -a is parsed as the dative case suffix rather than as the onset of a predicate.

In Sentence (4) (Figure 2b), the prosodic boundary after *balkon* licenses a completely different syntactic structure. *Balkon* is parsed as the clausal subject DP, and *açıktı* functions as the sentential predicate. The pause before the predicate prevents *balkon* from being analyzed as a dative-marked PP, and instead yields a subject–predicate structure: [TP [DP balkon] [T’ [PredP açık-tı]]]. Thus, prosodic phrasing determines whether *balkon* is interpreted as (i) a dative-marked goal argument of a motion verb or (ii) a simple subject DP. The two structures correspond to entirely distinct event representations, and prosodic boundary placement is the primary cue that supports disambiguation, although a full acoustic validation of all stimuli remains necessary.

For this representative item pair, the supplementary acoustic inspection showed that the boundary-marked sentence, *Balkon, açıktı*, had a longer total duration and a greater degree of voice breaks than the non-boundary sentence, *Balkona çıktı*. Mean F0 was highly similar across the two productions, suggesting that the contrast in this example was more strongly reflected in temporal and pausal organization than in global pitch level. These findings are reported descriptively in Supplementary Table S1 and should not be interpreted as systematic acoustic validation of the complete stimulus set.

### 2.3. Reliability and Validity

To evaluate the reliability and validity of the PCT, several complementary analyses were performed. Internal consistency reliability was examined through Cronbach’s alpha to determine the degree of homogeneity among items. It is important to note that the PCT is designed to assess prosodic comprehension as a multidimensional construct comprising three partially dissociable mechanisms; accordingly, moderate rather than high internal consistency is theoretically expected, particularly at the subscale level.

Construct validity was assessed using principal component analysis (PCA) with varimax rotation to identify the underlying dimensional structure of the PCT. We note that this approach constitutes PCA-based dimension reduction rather than common factor analysis (EFA) in the strict psychometric sense; this distinction is acknowledged as a limitation, and confirmatory factor analysis in an independent sample is recommended as a next step. Sampling adequacy was confirmed by the Kaiser–Meyer–Olkin measure (KMO = 0.74), and Bartlett’s test of sphericity (χ^2^(496) = 2593.16, *p* < 0.001) verified that the data were appropriate for factoring.

Convergent validity was assessed by correlating PCT total scores with MoCA-TR scores using Pearson’s correlation. The rationale is that both measures draw on cognitive capacities, including attention, working memory, and executive functions, which are known to support auditory–prosodic integration. A meaningful positive correlation would therefore be expected in a healthy sample, providing convergent evidence that the PCT captures aspects of cognitive-linguistic functioning. Results reflect convergent validity evidence in a healthy normative sample and do not constitute criterion validity or diagnostic accuracy evidence for prosodic impairment in clinical populations.

Content validity was established through expert judgment by five professionals (two associate professors and three full professors) specializing in neurology, otolaryngology, audiology, and speech–language pathology.

### 2.4. Study Procedures

The evaluation was conducted in a quiet, distraction-free room with one participant at a time. The auditory stimuli were presented using a sound source positioned directly in front of the participant to ensure equal bilateral auditory input. Audio files were played at a fixed intensity of 70 dB SPL, with the source located 30 cm from the participant’s head. Each sentence was accompanied by two printed visual stimuli (on 300 g matte canvas paper to prevent glare), representing the two possible interpretations of the sentence. These visuals were placed vertically on an adjustable stand to facilitate comfortable viewing at eye level.

Before the experimental task began, participants completed a structured learning phase designed to familiarize them with the prosodic contrast of interest. In this phase, each participant was exposed to two learning sessions, during which both sentences from a minimal prosodic pair were presented together. The prosodic difference between the two sentences was explicitly explained to the participant, and they were asked to choose the picture that best matched each sentence’s intended meaning. Corrective feedback was provided during this phase, and participants were required to demonstrate a clear understanding of the contrast by selecting the correct image for both sentences. Only participants who successfully completed the learning phase were allowed to proceed to the experimental trials.

Participants were exposed to 32 test items. Both sentences from each pair were used in the experiment, but each participant heard only one sentence per trial, presented in randomized order across all items. This ensured that sentence meaning was interpreted independently on each trial. In accordance with the sentence–picture matching paradigm, participants were asked to listen attentively and select the picture that best matched the meaning of the single sentence they heard based solely on prosodic cues. To maintain ecological validity and reduce the risk of learning effects, each sentence was presented only once without repetition.

To control for positional response biases, picture placement (left/right) was randomized across items and counterbalanced across participants. All participants received the same fixed order of auditory stimuli, but the side on which the correct image was presented was systematically varied to reduce guessing strategies (e.g., consistently choosing the left image).

Responses were binary coded as correct (1) or incorrect (0), and total scores were calculated as the sum of correct responses. During this session, the participant was shown a visual pair and given 10 s to examine the images. Then, both interpretations of the sentence “Uyuz Ahmet’e, geldi” were explained. For the sentence “Uyuz, Ahmet’e geldi”, it was clarified that “*uyuz*” referred to a contagious skin disease that had infected *Ahmet*. For “Uyuz Ahmet’e, geldi”, it was explained that “*uyuz*” was used as a slang adjective describing an unpleasant person, modifying the referent “*Ahmet*”.

Following the trial, participants were told they would see two images, be given 10 s to examine them silently, then hear a single sentence. Based on the auditory–prosodic cues alone, they were to point to the image corresponding to the meaning of that sentence. The full evaluation session, including the PCT and MoCA-TR, lasted approximately one hour per participant.

### 2.5. Statistical Analysis

All statistical analyses were conducted using IBM SPSS Statistics 26.0. The Kolmogorov–Smirnov test confirmed normal distribution of the data (*n* = 440). Accordingly, parametric procedures were employed. Descriptive statistics were computed for all variables. Cronbach’s alpha was calculated to assess internal consistency reliability. Construct validity was examined via PCA-based dimension reduction using varimax rotation; three components were retained based on the theoretical framework underlying the PCT (11 components showed eigenvalues greater than 1; however, retention was guided primarily by the theoretical framework underlying the PCT, together with component interpretability). A significance level of *p* < 0.05 was adopted. Convergent validity was evaluated through Pearson correlation (with 95% confidence intervals) between PCT total scores and MoCA-TR scores. Pearson correlations between PCT total scores and demographic variables (age, education level) were additionally computed to examine potential confounders. To further examine whether the association between PCT total scores and MoCA-TR scores was independent of demographic influences, a partial correlation analysis was conducted controlling for age and education level. A significance level of *p* < 0.05 was adopted for all analyses.

## 3. Results

The reliability study included 440 participants, the majority of whom had a university education (52.73%), while 28.64% had completed high school, and smaller percentages had finished middle school (9.09%) and primary school (9.55%). Most participants were monolingual (91.59%), and the gender distribution was nearly equal, with 50.45% female and 49.55% male participants. Analysis of participant ages revealed that individuals in the healthy control group ranged from 55 to 65 years old, with a mean age of 60.6 years (SD = 5.26).

Internal consistency analysis (Table 3) yielded an overall Cronbach’s alpha of 0.73 for the full 32-item scale. When individual items were systematically removed, alpha values remained stable in the range of 0.716 to 0.733, indicating that no single item disproportionately influenced overall reliability. Corrected item–total correlations ranged from 0.030 to 0.371. Items with lower item–total correlations (e.g., Items 3, 7, and 25) likely reflect the linguistic properties of specific prosodic contrasts rather than poor item quality; importantly, removing these items did not meaningfully increase internal consistency. Taken together, the full-scale reliability is acceptable, consistent with the multidimensional nature of the construct.

Subscale-level internal consistency varied considerably, with Cronbach’s alpha values of 0.25 (In situ Prosody), 0.45 (Focus–Topic Marking), and 0.59 (Pragmatic Disambiguation). These values are below conventional thresholds for clinical measurement, and this is acknowledged as an important limitation of the current version of the PCT. Each subscale is composed of items targeting a distinct type of prosodic processing that does not necessarily converge on a single latent dimension; accordingly, high internal consistency is not expected on theoretical grounds. The subscale alpha values should therefore be interpreted as reflecting the heterogeneity of the construct rather than as indicators of poor item quality. Nevertheless, subscale-level scores should be used and interpreted with caution in the current version of the PCT, and future item development aimed at improving subscale homogeneity is warranted.

According to Table 4, the Pearson correlation between PCT total scores and MoCA-TR scores was statistically significant (r = 0.23, *p* < 0.001, 95% CI [0.14, 0.32]), indicating a small-to-medium positive effect [46]. This result indicates that individuals with higher global cognitive performance also exhibited better prosodic comprehension performance. These findings support the convergent validity of the PCT, consistent with evidence that auditory–prosodic integration draws on domain-general cognitive resources including attention, working memory, and executive functions [46,47]. Additionally, age showed a significant negative correlation with PCT total scores (r = −0.25, *p* < 0.001, 95% CI [−0.33, −0.17]), indicating that older participants scored lower on average. Education level showed a significant positive correlation (r = 0.18, *p* < 0.001, 95% CI [0.09, 0.26]), indicating that participants with higher educational attainment performed better. These demographic associations are consistent with the normative literature on cognitive-linguistic test performance and should be considered when interpreting individual PCT scores. These results should be interpreted as convergent validity evidence in a healthy normative sample; cut-off scores for clinical use should be established in future studies with patient populations.

To examine whether the association between PCT total scores and MoCA-TR scores was influenced by demographic variables, a partial correlation analysis was conducted controlling for age and education level. The association remained statistically significant, although small in magnitude, partial r = 0.195, *p* < 0.001, 95% CI [0.101, 0.286], *n* = 420. This finding suggests that the relationship between global cognitive functioning and prosodic comprehension was not fully explained by age or educational differences in the present sample.

Table 5 presents the results of the PCA-based dimension reduction conducted on PCT items. Sampling adequacy was confirmed by the Kaiser–Meyer–Olkin measure (KMO = 0.74), and Bartlett’s test of sphericity was significant (χ^2^(496) = 2593.16, *p* < 0.001), indicating that the data were appropriate for component extraction. Three components were retained based on the a priori theoretical framework underlying the PCT. Of the 32 items, 11 components showed eigenvalues greater than 1 in the initial solution; however, following methodological recommendations for PCA in scale development contexts, the three-component solution was selected on theoretical grounds rather than by the eigenvalue-greater-than-1 criterion alone. The items clustered into three interpretable components consistent with the theoretical design: In situ Prosody, Focus–Topic Marking, and Pragmatic Disambiguation. The three components collectively accounted for 28.2% of the total variance. This level of explained variance is modest and reflects the heterogeneous and multi-mechanistic nature of prosodic comprehension in Turkish; it is a common outcome for tasks involving subtle cues across dissociable linguistic dimensions and should be interpreted cautiously and viewed as preliminary. Factor loadings showed substantial variability across items, with the Pragmatic Disambiguation component demonstrating comparatively higher loadings.

Table 6 presents the conceptual subscale organization of the PCT, aligned with the component patterns observed in the PCA (Table 5). Cronbach’s alpha coefficients for each subscale are reported alongside item assignments. The overall internal consistency of the full 32-item scale was 0.73. The expert panel consisted of five professionals: one neuropsychologist, two neurologists, and two speech–language therapists.

## 4. Discussion

The present study reported the development and initial psychometric evaluation of the Prosodic Comprehension Test (PCT) in a sample of 440 neurologically healthy native Turkish-speaking adults. The findings provide preliminary support for the PCT as a linguistically grounded instrument for assessing prosodic comprehension in Turkish.

### 4.1. Sample Characteristics and Normative Considerations

The sample demonstrated a predominantly high-education profile, with more than half of the participants holding a university degree. This distribution aligns with recruitment patterns commonly observed in normative linguistic studies but also suggests that the normative scores obtained may reflect enhanced metalinguistic awareness associated with higher educational attainment. Future studies should therefore include participants with a wider range of educational backgrounds to establish representative norms. The sample consisted primarily of monolingual Turkish speakers (91.6%), which strengthens the internal validity of the normative dataset. Gender distribution was nearly balanced. Notably, age showed a significant negative correlation with PCT total scores (r = −0.25, *p* < 0.001), suggesting that younger participants performed better on average, possibly reflecting the broader literature on age-related declines in processing speed and cognitive flexibility. Education level showed a significant positive correlation with PCT scores (r = 0.18, *p* < 0.001), consistent with effects documented for other higher-order language measures. These associations underscore the importance of stratifying normative data by age group and education level in future studies.

### 4.2. Item-Level Performance

Item-level analyses (Table 3) indicated that PCT items demonstrated an overall accuracy range of 0.76 to 0.96, suggesting items of low-to-moderate difficulty appropriate for assessing prosodic comprehension in neurologically healthy adults. Corrected item–total correlations predominantly ranged between 0.20 and 0.35, which is expected for an instrument designed to capture partially dissociable mechanisms of prosodic interpretation rather than a strictly unidimensional construct. Items with relatively lower correlations likely reflect structural properties of Turkish where certain prosodic contrasts may be easier to resolve due to salient contextual or morphological cues.

### 4.3. Convergent Validity and Cognitive Associations

The significant positive correlation between PCT total scores and MoCA-TR scores (r = 0.23, *p* < 0.001, 95% CI [0.14, 0.32]) provides convergent validity evidence, indicating that prosodic comprehension performance covaries with global cognitive functioning in healthy adults. This association is theoretically consistent with evidence that auditory–prosodic integration relies on domain-general cognitive resources, including attention, working memory, and executive functions [47,48]. ROC analysis was not conducted, as MoCA-TR is not a reference standard for prosodic processing and the present study is limited to a healthy normative sample. Cut-off scores for clinical use should be established in future studies with patient populations.

### 4.4. Factor Structure and Internal Consistency

The PCA produced a three-component structure consistent with the theoretical framework proposing that prosodic comprehension involves multiple interacting mechanisms [49,50]. The total explained variance of 28.2% is modest, a common outcome for tasks involving subtle and heterogeneous prosodic cues across multiple linguistic dimensions. The emergence of three components corresponding to in situ prosody, focus–topic marking, and pragmatic disambiguation offers preliminary empirical support for the conceptual framework underlying the PCT.

The labeling of the three components reflects the a priori theoretical design of the PCT rather than post hoc interpretation of factor loadings. The In Situ Prosody label denotes items in which a prosodic boundary is present but does not force reanalysis of grammatical category or argument structure; the target response corresponds to the default syntactic parse, making these items a perceptual baseline for prosodic sensitivity [1,6]. The Focus–Topic Marking label applies to items in which prosodic boundary shift reassigns information-structural roles—specifically, which constituent carries contrastive focus or is topicalized—within an otherwise fixed SOV frame, consistent with the established Turkish prosody and information structure literature [27,28,29,30]. The Pragmatic Disambiguation label covers items in which prosodic boundary placement is the primary cue resolving morphological ambiguity, homophony, or referential underspecification between two syntactically well-formed but pragmatically incompatible parses [51,52]. Because item-to-component assignment was determined before data collection on theoretical grounds, the component labels are independent of the PCA output and should be evaluated against the linguistic literature rather than against factor loading magnitudes alone.

Among the three subscales, pragmatic disambiguation showed the strongest internal consistency (α = 0.59), likely reflecting its broader item pool and the central role of prosodic boundaries in resolving naturally occurring ambiguities in Turkish. In contrast, in situ prosody and focus–topic marking demonstrated lower reliability (α = 0.25 and α = 0.45, respectively). These low values stem from the smaller number of items per subscale, the inherently low variance in structures where prosodic shifts yield subtle interpretive differences, and the fact that each subscale targets a distinct mechanism rather than a homogeneous latent dimension. The in situ prosody subscale in particular functions as a baseline index of prosodic sensitivity rather than as a measure of a strongly cohesive construct. The KMO value (0.74) and significant Bartlett’s test (χ^2^(496) = 2593.16, *p* < 0.001) confirm data adequacy for component extraction, although relatively low communalities indicate a need for further item development in future versions.

A further theoretical consideration concerns the nature of pragmatic disambiguation itself. Unlike syntactic parsing or focus–topic marking, pragmatic interpretation does not arise from a single linguistic level in isolation. Rather, pragmatic meaning emerges through the integration of information distributed across multiple representational domains, including prosodic structure, morphosyntactic organization, lexical-semantic content, discourse context, and speaker intentions [48,49,53]. From this perspective, the heterogeneity observed within the Pragmatic Disambiguation subscale should be interpreted cautiously, but it should not be viewed only as a psychometric weakness. It also reflects the inherently integrative nature of pragmatic processing, in which listeners coordinate multiple linguistic and contextual cues to resolve ambiguity. This issue is particularly relevant for Turkish, where flexible word order, extensive morphological ambiguity, and context-sensitive interpretation frequently interact with prosodic boundary placement during comprehension [29,32]. Accordingly, the Pragmatic Disambiguation subscale was designed to sample naturally occurring ambiguity-resolution processes rather than to isolate a single homogeneous prosodic operation. For this reason, the total PCT score remains more psychometrically defensible than the individual subscale scores at this preliminary validation stage.

It should be acknowledged that PCA with varimax rotation, as employed here, represents dimension reduction rather than common factor analysis, and the three-component solution should be treated as exploratory and hypothesis-generating. Confirmatory factor analysis in an independent sample will be essential to test the proposed three-factor structure. Because no standardized Turkish instrument specifically assessing prosodic comprehension currently exists, external convergent validity could not be evaluated against a domain-specific reference measure. Therefore, MoCA-TR was used solely to provide convergent validity evidence regarding the relationship between prosodic comprehension performance and general cognitive-linguistic functioning in a neurologically healthy sample. Future studies should evaluate convergent validity using clinical group comparisons and modality-specific prosodic processing measures.

### 4.5. Limitations

Several limitations of the present study should be acknowledged. First, the PCT was validated solely in neurologically healthy adults. While this is a necessary first step for test development, it does not permit conclusions about clinical diagnostic accuracy for prosodic impairment or about the performance of individuals with stroke, aphasia, right-hemisphere damage, mild cognitive impairment, or other neurological conditions. Any clinical applications of the PCT should await targeted validation studies with patient populations.

Second, each test stimulus is presented only once, which increases trial-level measurement error. While single-trial presentation was chosen to reduce learning effects and maintain ecological validity, test–retest reliability and the effects of repeated exposure should be examined in future studies.

Third, the structured learning phase, while procedurally necessary to ensure comprehension of the task, may introduce an element of explicit strategy formation that partially reduces ecological validity. Future research should explore whether abbreviated or modified familiarization procedures can minimize this concern without compromising task comprehension.

Fourth, the PCA-based analyses employed here do not constitute full exploratory factor analysis in the common factor model sense. Confirmatory factor analysis in an independent and more demographically diverse sample is required to validate the proposed three-factor structure.

Fifth, the current sample was relatively homogeneous in terms of education and language experience (predominantly university-educated monolinguals), which may limit the generalizability of the normative scores. Future studies should include participants with more diverse educational and sociolinguistic profiles to establish broadly applicable norms.

Sixth, although an illustrative acoustic inspection of one representative item pair is provided in Supplementary Table S1, a comprehensive acoustic analysis confirming the absence of non-prosodic cues across all stimuli was not conducted in the present study.

Despite these limitations, the PCT offers a linguistically grounded and systematically developed instrument that addresses an important gap in Turkish clinical assessment by providing a controlled measure of prosodic comprehension sensitive to the syntactic, information-structural, and pragmatic dimensions of Turkish prosody. Given the multidimensional nature of prosodic comprehension, the PCT was not designed as a strictly unidimensional scale.

## 5. Conclusions and Further Perspectives

This study reported the initial validity and reliability findings of the Prosodic Comprehension Test (PCT), a newly developed instrument for evaluating sentence-level meaning shaped by the interaction between syntactic structure and prosodic boundary placement. The results suggest that the PCT is a promising tool for capturing prosodic comprehension in Turkish, a language in which prosody frequently supports the resolution of structural and pragmatic ambiguity.

The full-scale internal consistency was acceptable (α = 0.73), and PCA-based analyses revealed a three-component structure consistent with the theoretical framework. Convergent validity was supported by a statistically significant positive correlation with global cognitive functioning (r = 0.23, *p* < 0.001, 95% CI [0.14, 0.32]), representing a small-to-medium effect. These findings constitute an important first step in establishing the psychometric properties of the PCT in a normative adult sample.

From a clinical standpoint, the development of such an instrument is important because many communicative deficits in acquired neurological conditions emerge in the domain of prosody and pragmatic interpretation. By specifically targeting prosodic mechanisms that support syntactic parsing, information-structure marking, and pragmatic disambiguation, the PCT may ultimately assist clinicians in identifying comprehension difficulties that may go unnoticed in standard aphasia or cognitive-linguistic batteries—provided that future validation with clinical populations confirms its diagnostic utility.

Looking ahead, a research program is needed that integrates the PCT into a broader assessment framework including discourse-level prosodic tasks, neurocognitive measures capturing attention and working-memory contributions, and systematic testing with diverse clinical subgroups. Future studies should also prioritize test–retest reliability, confirmatory factor analysis in independent samples, acoustic validation of stimuli, and development of norms across a wider range of educational and demographic profiles. Because PCT items are dichotomously scored, future psychometric work would benefit from analytic approaches suited to binary data, including tetrachoric factor analysis, item response theory modeling, and reliability estimates such as McDonald’s omega and KR-20, in addition to test–retest assessment of temporal stability. Such an integrated approach will facilitate the development of a comprehensive model of prosodic processing in Turkish and enhance the utility of the PCT across research and clinical domains.

## Figures and Tables

**Table 1:** Demographic characteristics of the healthy participants in the PCT reliability study.

Category	Subcategory	*N*	%
Education Level	Primary School	42	9.55
	Middle School	40	9.09
	High School	126	28.64
	University	232	52.73
	Total	440	100
Language Status	Monolingual	403	91.59
	Bilingual	37	8.41
	Total	440	100
Gender	Female	222	50.45
	Male	218	49.55
	Total	440	100

**Table 2:** PCT Item Stimuli: Prosodic Minimal Pairs With Glosses and Translations.

#	Sentence (In Situ/Odd Items)	#	Sentence (Focus–Topic/Even Items)
**L**	**Uyuz# Ahmet’e geldi.** Uyuz, Ahmet-e gel-di. Scab Ahmet-DAT come-PF-3SG. ‘Ahmet is infected with scabies.’	**L**	**Uyuz Ahmet’e# geldi.** Uyuz Ahmet-e, gel-di. (He [null]) scab Ahmet-DAT come-PF-(3SG). ‘He came to the idiot Ahmet.’
**1**	**Yaşlı# ağacın altında uyuyordu.** Yaşlı, ağaç-ın alt-ı-nda uyu-yor-du. The old man tree-GEN under-3SG.POSS-LOC sleep-IMPF-PF-3SG. ‘The old man was sleeping under the tree.’	**2**	**Yaşlı ağacın# altında uyuyordu.** Yaşlı ağaç-ın, alt-ı-nda uyu-yor-du. (He [null]) old tree-GEN under-3SG.POSS-LOC sleep-IMPF-PF-(3SG). ‘He was sleeping under the old tree.’
**3**	**Bu# okulda başarılı olmuştu.** Bu, okul-da başarılı ol-muş-tu. He school-LOC successful be-EV/PF-3SG. ‘He was successful in school years.’	**4**	**Bu okulda# başarılı olmuştu.** Bu okul-da, başarılı ol-muş-tu. (He [null]) this school-LOC successful be-EV/PF-(3SG). ‘He was successful in this school.’
**5**	**Oku da adam ol# baban gibi eşek olma.** Oku da adam ol, baba-n gibi eşek ol-ma. Get education man be like father-2SG.POSS ass be-NEG. ‘Be educated and don’t be an ass like your father.’	**6**	**Oku da adam ol baban gibi# eşek olma.** Oku da adam ol baba-n gibi, eşek ol-ma. Get education man be like father-2SG.POSS ass be-NEG. ‘Get education like your father, don’t be an ass.’
**7**	**Güzel# konuş bakalım.** Güzel, konuş bakalım. ‘Good, let me hear you talk.’	**8**	**Güzel konuş bakalım!** Güzel konuş bakalım. ‘Talk niceties!’
**9**	**Hasta# doktora seslendi.** Hasta, doktor-a seslen-di. Patient doctor-DAT call-PF-3SG. ‘The patient called out to the doctor.’	**10**	**Hasta doktora# seslendi.** Hasta doktor-a, seslen-di. (He [null]) sick doctor-DAT call-PF-(3SG). ‘He called out to the sick doctor.’
**11**	**Yaşlı# doktora bir şeyler anlatıyordu.** Yaşlı, doktor-a bir şey-ler anlat-ıyor-du. The old man doctor-DAT something-PL tell-IMPF-PF-3SG. ‘The old man was telling the doctor something.’	**12**	**Yaşlı doktora# bir şeyler anlatıyordu.** Yaşlı doktor-a, bir şey-ler anlat-ıyor-du. (He [null]) old doctor-DAT something-PL tell-IMPF-PF-(3SG). ‘He was telling the old doctor something.’
**13**	**Genç# adama bakıyordu.** Genç, adam-a bak-ıyor-du. The youth man-DAT look-IMPF-PF-3SG. ‘The youngster was looking at the man.’	**14**	**Genç adama# bakıyordu.** Genç adam-a, bak-ıyor-du. (She [null]) young man-DAT look-IMPF-PF-(3SG). ‘She was looking at the young man.’
**15**	**Hasta# çocuğuna gülümsedi.** Hasta, çocuk-u-na gülümse-di. Patient child-3SG.POSS-DAT smile-PF-3SG. ‘The patient smiled at his child.’	**16**	**Hasta çocuğuna# gülümsedi.** Hasta çocuk-u-na, gülümse-di. (He [null]) sick child-GEN-DAT smile-PF-(3SG). ‘He smiled at his sick child.’
**17**	**Dikkatli ol. İğne kırık. Koluna batmasın.** Dikkatli ol. İğne kırık. Kol-u-na bat-ma-sın. Be careful. Needle broken. Arm-2SG.POSS-DAT stick-NEG-OPT-(3SG). ‘Be careful. The needle’s broken. Don’t stick it in your arm.’	**18**	**Dikkatli ol. İğne# kırık koluna batmasın.** Dikkatli ol. İğne, kırık kol-un-a bat-ma-sın. Be careful. Needle broken arm-2SG.POSS-DAT stick-NEG-OPT-3SG. ‘Be careful. Don’t stick the needle in your broken arm.’
**19**	**Kadın# itfaiyeciye bağırdı.** Kadın, itfaiyeci-ye bağır-dı. Woman fireman-DAT shout-PF-3SG. ‘The woman shouted at the fireman.’	**20**	**Kadın itfaiyeciye# bağırdı.** Kadın itfaiyeci-ye, bağır-dı. (He [null]) female firefighter-DAT shout-PF-(3SG). ‘He shouted at the female firefighter.’
**21**	**Hırsız# çocuğu dövdü.** Hırsız çocuk-u döv-dü. Thief kid-ACC beat-PF-3SG. ‘The thief beat the kid.’	**22**	**Hırsız çocuğu# dövdü.** Hırsız çocuk-u, döv-dü. (He [null]) thief kid-ACC beat-PF-(3SG). ‘He beat the thief boy.’
**23**	**Balkona çıktı.** Balkon-a çık-tı. (He [null]) balcony-DAT went.out-PF-(3SG). ‘He went out to the balcony.’	**24**	**Balkon# açıktı.** Balkon, açık-tı. Balcony open-P.COP-3SG. ‘The balcony was open.’
**25**	**Yer# eserdi.** Yer, es-er-di. The place wind-AOR-P.COP-3SG. ‘The place was windy.’	**26**	**Yere serdi.** Yer-e ser-di. (He [null]) ground-DAT roll out-PF-(3SG) (rug [null obj]). ‘He rolled out the rug to the ground.’
**27**	**Bu gece kızıl# ay var.** Bu gece kızıl ay var. Tonight red moon existent (-GM). ‘There is a red moon tonight.’	**28**	**Bu gece Kızılay var.** Tonight Kızılay (charity org.) existent (-GM). ‘There is Kızılay tonight.’
**29**	**Sevgilim# hayal et.** Sevgili-m hayal, et. Darling-1SG.POSS imagine-(2SG). ‘My darling, imagine.’	**30**	**Sevgilim hayalet.** Sevgili-m hayalet. Darling-1SG.POSS ghost-(GM). ‘My girlfriend is a ghost.’
**31**	**Adama# normal görünüyor.** Adam-a, normal görün-üyor. (He [null]) man-DAT normal look-IMPF-(3SG). ‘He looks normal to one.’	**32**	**Adam# anormal görünüyor.** Adam, anormal görün-üyor. Man abnormal look-IMPF-3SG. ‘The man has an abnormal appearance.’

*Note*. # = prosodically marked boundary. L = Learning Phase item pair (presented before experimental trials). DAT = Dative. PF = Perfective. 3SG = Third person singular. POSS = Possessive. LOC = Locative. IMPF = Imperfective. EV/PF = Evidential/Perfective. NEG = Negative. 2SG = Second person singular. PL = Plural. GEN = Genitive. OPT = Optative. ACC = Accusative. P.COP = Past copula. AOR = Aorist. GM = Generalizing modality. 1SG = First person singular. In Turkish, third-person singular subjects are indicated by the absence of a person marker. Bold = items of the PCT.

**Table 3:** Item analysis of the PCT.

	Mean ± SD	Scale Mean if Item Deleted	Corrected Item–Total Correlation	Cronbach’s Alpha if Item Deleted
Item 1	0.76 ± 0.43	27.27	0.201	0.728
Item 2	0.89 ± 0.31	27.14	0.187	0.727
Item 3	0.9 ± 0.3	27.13	0.113	0.731
Item 4	0.87 ± 0.34	27.16	0.278	0.722
Item 5	0.84 ± 0.55	27.19	0.205	0.731
Item 6	0.86 ± 0.35	27.17	0.212	0.726
Item 7	0.91 ± 0.28	27.12	0.118	0.731
Item 8	0.84 ± 0.37	27.19	0.209	0.727
Item 9	0.86 ± 0.35	27.17	0.231	0.725
Item 10	0.85 ± 0.35	27.18	0.253	0.724
Item 11	0.89 ± 0.32	27.14	0.169	0.729
Item 12	0.93 ± 0.26	27.1	0.056	0.733
Item 13	0.89 ± 0.31	27.14	0.339	0.719
Item 14	0.9 ± 0.31	27.13	0.329	0.72
Item 15	0.86 ± 0.35	27.17	0.296	0.721
Item 16	0.85 ± 0.35	27.18	0.329	0.719
Item 17	0.9 ± 0.3	27.13	0.191	0.727
Item 18	0.91 ± 0.29	27.12	0.275	0.723
Item 19	0.9 ± 0.3	27.13	0.2	0.727
Item 20	0.91 ± 0.29	27.12	0.349	0.719
Item 21	0.81 ± 0.4	27.22	0.136	0.732
Item 22	0.9 ± 0.3	27.13	0.286	0.722
Item 23	0.89 ± 0.31	27.14	0.289	0.722
Item 24	0.82 ± 0.38	27.21	0.227	0.726
Item 25	0.95 ± 0.21	27.08	0.03	0.733
Item 26	0.82 ± 0.39	27.21	0.364	0.716
Item 27	0.84 ± 0.37	27.19	0.366	0.716
Item 28	0.96 ± 0.2	27.07	0.13	0.73
Item 29	0.91 ± 0.29	27.12	0.322	0.72
Item 30	0.91 ± 0.29	27.12	0.259	0.724
Item 31	0.88 ± 0.33	27.15	0.371	0.717
Item 32	0.84 ± 0.37	27.19	0.321	0.719

**Table 4:** Convergent validity: Pearson correlation between PCT and MoCA-TR scores.

Analysis Type	Metric	Value	95% CI (Lower–Upper)	*p*	Interpretation
Pearson Correlation (PCT–MoCA-TR)	***r***	0.231	[0.14, 0.32]	<0.001	Small-to-medium positive effect
Partial Correlation (PCT–MoCA-TR [controlling for age and education])	***r***	0.195	[0.101, 0.286]	<0.001	Small positive association

*Note*. Pearson correlation was computed to examine the linear association between PCT total scores and MoCA-TR scores. Results reflect convergent validity in a healthy adult normative sample and do not constitute criterion validity or diagnostic accuracy evidence for prosodic impairment in clinical populations. Partial correlation was based on complete cases for age and education.

**Table 5:** PCA-based dimension-reduction and internal consistency results for the PCT.

Factor/Item	Factor Loading	Communality (h^2^)	Eigenvalue	% Variance	Cumulative %
Factor 1: In Situ Prosody			4.82	15.1	15.1
Item 1	0.48	0.23			
Item 3	0.15	0.08			
Item 9	0.27	0.07			
Item 11	0.20	0.10			
Item 13	0.15	0.06			
Item 15	−0.25	0.06			
Item 19	0.22	0.11			
Item 21	−0.34	0.12			
α = 0.25					
Factor 2: Focus–Topic Marking			2.41	7.5	22.6
Item 2	0.20	0.05			
Item 4	0.21	0.04			
Item 10	0.18	0.03			
Item 12	0.04	0.01			
Item 14	0.24	0.12			
Item 16	0.10	0.02			
Item 20	0.11	0.02			
Item 22	0.12	0.02			
α = 0.45					
Factor 3: Pragmatic Disambiguation			1.76	5.5	28.2
Item 5	0.73	0.54			
Item 6	−0.16	0.03			
Item 7	−0.09	0.01			
Item 8	−0.28	0.09			
Item 17	−0.10	0.01			
Item 18	0.15	0.02			
Item 23	0.18	0.03			
Item 24	−0.21	0.04			
Item 25	0.03	0.01			
Item 26	−0.44	0.20			
Item 27	−0.32	0.16			
Item 28	0.06	0.01			
Item 29	0.07	0.02			
Item 30	−0.25	0.06			
Item 31	0.24	0.13			
Item 32	−0.33	0.11			
α = 0.59					

*Note*. Kaiser–Meyer–Olkin measure of sampling adequacy = 0.74. Bartlett’s test of sphericity: χ^2^(496) = 2593.16, *p* < 0.001. Extraction method: Principal Component Analysis (PCA). Rotation method: Varimax. Items were grouped according to theoretical categorization (In Situ Prosody, Focus–Topic Marking, Pragmatic Disambiguation); factor loadings are provided for descriptive purposes only. Total explained variance = 28.2%. PCA-based extraction represents dimension reduction rather than common factor analysis; the three-component solution is exploratory and hypothesis-generating. Confirmatory factor analysis in an independent sample is recommended. Note: 11 components showed eigenvalues > 1 in the initial solution; three components were retained on a priori theoretical grounds rather than by the eigenvalue-greater-than-1 criterion.

**Table 6:** Conceptual subscale organization of the PCT and reliability coefficients.

Factor	Description	Items	Cronbach’s α *
Factor 1—In situ Prosody	Prosodic boundary does not shift the interpretation; meaning is resolved with neutral, in situ prominence.	1, 3, 9, 11, 13, 15, 19, 21	0.25
Factor 2—Focus–Topic Marking	Prosodic prominence marks contrastive focus or topicalization; boundary placement changes the information structure.	2, 4, 10, 12, 14, 16, 20, 22	0.45
Factor 3—Pragmatic Disambiguation	Interpretation depends on prosodic boundary–driven pragmatic inference; boundary shift alters the intended meaning.	5, 6, 7, 8, 17, 18, 23, 24, 25, 26, 27, 28, 29, 30, 31, 32	0.59
Total Scale	All 32 PCT items	1–32	0.73

*Note*. Cronbach’s α values for subscales are below conventional thresholds for standalone clinical measurement (α = 0.25, 0.45, 0.59) and should be interpreted cautiously given the small item counts per subscale and the theoretically heterogeneous nature of each subscale’s item pool. The full-scale score (α = 0.73, 32 items) is recommended as the primary reliability index.

## Data Availability

The data and analysis code supporting the findings of this study are not publicly available due to institutional data sharing restrictions. We are actively working to incorporate reproducible research practices into future studies, including the use of open-source analysis pipelines and revised informed consent procedures that support data sharing.

## Supplementary Materials

The following supporting information can be downloaded at https://www.mdpi.com/article/10.3390/audiolres16040099/s1, Table S1: Illustrative acoustic comparison of one representative PCT item pair.
