# Supplementary material for: Development and Preliminary Validation of the Turkish Prosodic Comprehension Test (PCT)
Source: Audiol Res. 2026 Jun 30;16(4):99. doi: 10.3390/audiolres16040099 (PMC13397981; doi:10.3390/audiolres16040099)
Supplement: Supplementary file 1 [file audiolres-16-00099-s001.zip › audiolres-4321208-supplementary.pdf]

A preliminary acoustic inspection was conducted for one representative item pair, namely *Balkona çıktı* ("He went out to the balcony") and *Balkon, açıktı* ("The balcony was open"), to illustrate how prosodic boundary placement is reflected acoustically. This analysis was not intended as a full acoustic validation of the stimulus set, but rather as a descriptive example of the acoustic contrast underlying one item pair.

Table S1. Illustrative acoustic comparison of one representative PCT item pair

Acoustic parameter	*Balkon, açıktı*	*Balkona çıktı*	Difference
**Duration, s**	1.868	1.441	-0.427
**Median F0, Hz**	116.33	126.79	+10.46
**Mean F0, Hz**	118.70	119.23	+0.53
**F0 range, Hz**	75.48	61.31	-14.17
**Locally unvoiced frames, %**	44.32	38.19	-6.13 pp
**Voice breaks, n**	4	3	-1
**Degree of voice breaks, %**	34.46	23.13	-11.33 pp
**Voice-break duration, s**	0.644	0.333	-0.310
**Jitter local, %**	3.41	2.85	-0.57 pp
**Shimmer local, %**	11.15	9.63	-1.52 pp
**NHR**	0.257	0.221	-0.036
**HNR, dB**	8.88	9.40	+0.52

The acoustic inspection indicated that the boundary-marked sentence (*Balkon, açıktı*) had a longer total duration and greater voice-break degree than the non-boundary sentence (*Balkona çıktı*). In contrast, mean F0 was highly similar across the two productions. These descriptive findings suggest that the contrast between the two items was primarily reflected in temporal and pausal organization rather than in global pitch

level. Because this analysis was limited to a single illustrative item pair and did not systematically examine all stimuli, it should not be interpreted as full acoustic validation of the PCT stimulus set.
